# The clinical application of metagenomic next-generation sequencing in sepsis of immunocompromised patients

**DOI:** 10.3389/fcimb.2023.1170687

**Published:** 2023-04-24

**Authors:** Xingxing Li, Shunda Liang, Dan Zhang, Miao He, Hong Zhang

**Affiliations:** Department of Emergency Medicine, The First Affiliated Hospital of Anhui Medical University, Anhui, Hefei, China

**Keywords:** metagenomic next-generation sequencing, sepsis, immunocompromised patients, diagnostic, sensitivity, clinical effect

## Abstract

**Background:**

Metagenomic next-generation sequencing (mNGS) was commonly applied given its ability to identify and type all infections without depending upon culture and to retrieve all DNA with unbiasedness. In this study, we strive to compare outcomes of mNGS with conventional culture methods in adults with sepsis, investigate the differences between the immunocompromised and control group, and assess the clinical effects of mNGS.

**Methods:**

In our study, 308 adult sepsis patients were included. We used both mNGS and conventional culture methods to analyze diagnostic results, pathogens, and sample types. The correlation between some laboratory tests and the frequency of pathogens by groups was also analyzed. Furthermore, the clinical impacts of mNGS were estimated.

**Results:**

308 samples were assigned to an immunocompromised group (92/308,29.9%) and a control group (216/308,70.1%). There was the sensitivity of mNGS considered greater than that of the culture method in all samples (88.0% vs 26.3%; P <​ 0.001), in the immunocompromised group (91.3% vs 26.1%; P <​ 0.001), and the control group (86.6% vs 26.4%; P <​ 0.001), particularly in all sample types of blood (P <​ 0.001), BALF (P <​ 0.001), CSF (P <​ 0.001), sputum (P <​ 0.001) and ascitic fluid (P = 0.008). When examining the mNGS results between groups, Pneumocystis jirovecii (P < 0.001), Mucoraceae (P = 0.014), and Klebsiella (P = 0.045) all showed significant differences. On the whole, mNGS detected more pathogens than culture methods (111 vs 25), found 89 organisms that were continuously overlooked in entire samples by culture methods, and showed a favorable positive clinical effect in 76.3% (235 of 308) of patients. In 185 (60.1%) patients, mNGS prompted a modification in the course of management, which included antibiotic de-escalation in 61(19.8%) patients.

**Conclusions:**

The research discovered that mNGS was more sensitive than the culture method, particularly in samples of blood, BALF, CSF, sputum, and ascitic fluid. When examining the mNGS results, Pneumocystis jirovecii and Mucoraceae were the pathogens seen more commonly in immunocompromised patients with sepsis, which required more attention from clinicians. There was a substantial benefit of mNGS in enhancing the diagnosis of sepsis and advancing patient treatment.

## 1 Introduction

Sepsis is a serious medical problem that affects people all over the world and is accompanied by a high incidence of morbidity and mortality. Despite the significant improvements in the care of immunocompromised patients over the past few decades, sepsis remains to be the main reason for death in this population. The diagnosis and management of severe infections may be more problematic due to patients’ limited capacity to manifest the clinical symptoms that normally accompany sepsis, but essential to salvaging the patient outcomes (McCreery et al., 2020). The microbiology laboratory, as the first line of pathogen detection, contributes significantly to controlling infections by microscopy, culture, classification, drug sensitivity, and other means (Zhou et al., 2016). Nevertheless, pathogens can be undiagnosed in roughly 60% of samples as a result of the limitations of molecular diagnostics and genotyping approaches (Ewig et al., 2002; van Gageldonk-Lafeber et al., 2005; Schlaberg et al., 2017). When microorganisms fail to be identified in time, broad-spectrum antibiotics may be used unnecessarily, which leads to the development of resistance and an increase in medical expenses (Miao et al., 2018).

An unbiased molecular technique called metagenomics next-generation sequencing (mNGS) may concurrently identify bacteria, viruses, fungi, and parasites in clinical specimens by detecting all of their DNA and/or RNA content (Chiu and Miller, 2019). Previous research has shown that mNGS, distinguished by high accuracy, extreme sensitivity, and rapidly detectable time, may identify pathogens in an array of specimens (Chiu and Miller, 2019; Wilson et al., 2019; Chen et al., 2021; Gu et al., 2021). It has important benefits for identifying pathogens that cause severe infections, mixed infections, and uncommon and novel pathogen infections in immunocompromised patients (Chiu and Miller, 2019). Whereas, it is unknown whether mNGS is effective in the etiological diagnosis and management of immunocompromised individuals with sepsis.

Thus, we analyzed sensitivities between the mNGS method and the conventional culture method to identify pathogens and evaluated the effects of mNGS detection outcomes on the diagnosis and management of immunocompromised patients with sepsis in our study.

## 2 Materials and methods

### 2.1 Enrolled patients

A total of 308 adult patients with sepsis were enrolled in this study at The First Affiliated Hospital of Anhui Medical University in Anhui, China, from January 2021 to December 2021. The diagnosis of sepsis met the diagnostic criteria set out by the Society of Critical Care Medicine (SCCM) and the European Society of Intensive Care Medicine (ESICM) (Singer et al., 2016). According to immunological status, the patients were split into an immunocompromised group and a control group.

Based on the previous study, the following definition of the immunocompromised state (Hill, 2020) was used: ① hematological malignancies; ② solid organ transplantation or hematopoietic stem cell transplantation (HCT); ③ solid tumors recently treated with chemotherapeutic agents; ④ primary immunodeficiency disease; ⑤ HIV infection with a CD4+ T-lymphocyte count <200 cells/ul; ⑥ taken immunosuppressants, biological immunomodulators, and anti-rheumatic drugs (e.g., methotrexate, cyclophosphamide, and cyclosporin); ⑦ taken 20 mg of glucocorticoids daily for more than 14 days (or 700 mg of prednisolone cumulatively, or equivalent doses of other corticosteroids).

### 2.2 Clinical data and sample collection

Two experienced resident physicians collected clinical data independently. Baseline data from electronic medical records were obtained, including demographic characteristics, past illness history, immunocompromised state, laboratory test, treatment procedure, and prognosis. A group of three senior doctors reviewed the data.

Samples from the infected location were collected from sepsis patients by standard procedures. Blood samples were obtained if the primary infection site was not known or its sample was not available. Each sample of blood, BALF, or urine contained a minimum of 5 ml, and each sample of CSF, sputum or other sterile liquid had at least 3 ml. Pathogen detection was carried out on entire specimens using mNGS and conventional culture methods simultaneously.

### 2.3 Etiological diagnosis

The conventional culture methods include bacterial culture, fungal culture, acid-fast bacterial culture, and blood culture. Blood cultures contain aerobic and anaerobic cultures. We performed conventional culture and mNGS methods according to the patient’s medical conditions, and finally selected cases with the same specimens for examination to be included in this study. However, it was not required that all types of cultures be performed on each patient. Microbial culture and automated microbial identification systems were utilized.

Following standard operating procedures, nucleic acid extraction, nucleic acid fragmentation, end repair, end adenylation, primer ligation, and purification were performed from each sample to form a sequencing library using kits from an automated workstation (Amar et al., 2021). Libraries were assessed for quality using kits quantified by real-time PCR and loaded onto an illumina Nextseq CN500 sequencer for 75 cycles of single-end sequencing, producing approximately 20 million reads per library (Miller et al., 2019). Furthermore, peripheral hematopoietic cell specimens from healthy donors were used as negative controls simultaneously, and sterile deionized water was represented as non-template controls concurrently with each batch (Miller et al., 2019).

### 2.4 Bioinformatics analyses

All original sequence reads are eliminated by the bioinformatics analysis software for low-quality and complex reads, duplicate reads, reads shorter than 50bp, contamination reads, and human sequence data (Li and Durbin, 2009; Bolger et al., 2014). In the end, there were approximately 13,000 genomes included in the final database. The remaining sequence data were aligned to a microbial database (including bacteria, viruses, fungi, and parasites) designed by a technology company, which is comparable to the National Center for Biotechnology Information (NCBI) Nucleotide and Genome databases, to determine the species and relative abundance of pathogens. Pathogen lists were chosen based on three references: 1) Manual of Clinical Microbiology, 2) Johns Hopkins ABX Guide, and 3) clinical case reports or academic studies recently appeared in peer-reviewed publications. RPM-r was defined as the reads per million (RPM) of a particular organism in the clinical sample divided by the RPM of the negative control. If the RPM-r was ≥ 5, and the RPM for bacteria and fungi were more than 10 and 2 respectively, there was a reported positive detection for the certain pathogen (Miller et al., 2019; Zinter et al., 2019). A viral detection result was considered positive when three or more non-overlapping areas of the genome were covered.

### 2.5 Definition of clinical effect

In this study, the identification of pathogenic microorganisms was carried out independently by a group of three senior doctors. They made this diagnosis based on clinical manifestations, laboratory tests, mNGS results, imaging studies, and treatment adjustments from patients. Any disputes between clinicians are resolved by further discussion.

A positive effect was defined as the use of mNGS results to support etiology diagnosis and adjust the anti-infective management, including change in antibiotic treatment, antibiotic de-escalation, and continuation of the empirical antibiotic treatment. A negative effect was defined as the use of mNGS results to make a mistaken diagnosis resulting in unnecessary or inadequate antibiotic treatment. A negative mNGS result and an incorrect or insignificant mNGS result were deemed to have no clinical effect.

### 2.6 Statistic analysis

The SPSS 23.0 software was employed to statistically analyze the data. Normal distribution of continuous variables used the Kolmogorov-Smirnov test and measurement data in accord with normal distribution was performed as mean ± standard deviation. An independent sample t-test was utilized between groups. Non-normal distribution measurement data were represented as median (lower quartile, upper quartile), and we applied a nonparametric test for comparison between groups. Comparative analysis was carried out by Pearson’s χ2 test. P values below 0.05 were regarded as significant.

## 3 Results

### 3.1 Characteristics of patients and samples

**Figure 1:**
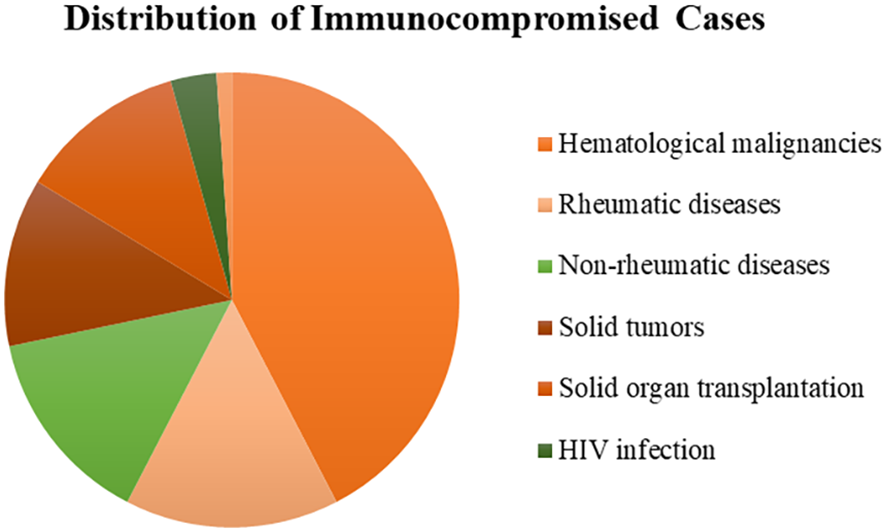
The distribution of immunocompromised patients. Most had hematological malignancies (42.3%), followed by rheumatic diseases (15.2%), non-rheumatic diseases (14.1%), solid tumors (12.0%), solid organ transplantation (12.0%), HIV infection (3.3%), and hematopoietic stem cell transplantation (1.1%). HIV, human immunodeficiency virus.

**Figure 2:**
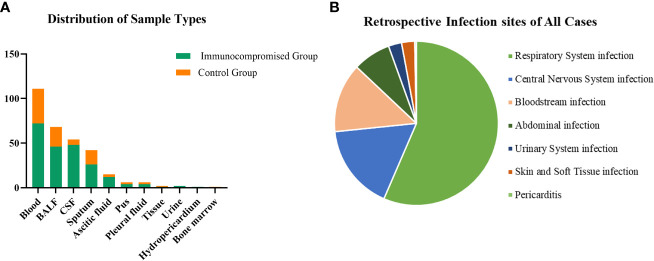
The distribution of sample types and infection sites. **(A)** In samples of this study, 36.0% were from the blood which was the most, 22.1% from BALF, 17.5% from CSF, and the others were from sputum (13.6%), ascitic fluid (4.9%), pus (1.9%), pleural fluid (1.9%), tissue (0.6%), urine (0.6%), hydropericardium (0.3%), bone marrow (0.3%). **(B)** Most infection sites were respiratory system infections (174/308,56.5%) and followed by central nervous system infections (52/308,16.9%), bloodstream infections (42/308, 13.6%), abdominal infections (23/308,7.5%), urinary system infections (8/308,2.6%), skin and soft tissue infections (8/308,2.6%), pericarditis (1/308,0.3%). CSF, cerebrospinal fluid; BALF, bronchoalveolar lavage fluid.

A total of 308 patients were enrolled in our study, of whom 92 patients were in the immunocompromised group and 216 patients were in the control group. The majority of patients in the immunocompromised group (n=92) had hematological malignancies (39/92, 42.3%), followed by rheumatic diseases (14/92,15.2%), non-rheumatic diseases with long-period glucocorticoid (13/92,14.1%), solid tumors recently treated with chemotherapeutic agents (11/92,12.0%), solid organ transplantation (11/92,12.0%), HIV infection with a CD4+ T-lymphocyte count < 200cells/ul (3/92,3.3%), and hematopoietic stem cell transplantation (HCT) (1/92,1.1%) (**Figure 1**). Blood made up the majority of our samples (111/308, 36.0%), followed by BALF (68/308, 22.1%), CSF (54/308, 17.5%), and sputum (42/308, 13.6%), as well as ascitic fluid (15/308, 4.9%), pus (6/308, 1.9%), pleural fluid (6/308, 1.9%), tissue (2/308, 0.6%), urine (2/308, 0.6%), hydropericardium (1/308, 0.3%), bone marrow (1/308, 0.3%) (**Figure 2A**). The majority of infectious sites were confirmed with the respiratory system (174/308,56.5%), followed by the central nervous system (52/308,16.9%), bloodstream (42/308, 13.6%), abdominal (23/308,7.5%), urinary system (8/308,2.6%), skin and soft tissue (8/308,2.6%), pericarditis (1/308,0.3%) (**Figure 2B**).

In **Table 1**, the fundamental clinical data about the patients is displayed. The immunocompromised patients were substantially younger than those in the control group, indicating that age could be a risk factor for sepsis in immunocompromised patients. Furthermore, the proportion of patients receiving glucocorticoids and blood products was statistically greater in the immunocompromised group than those in the control group, suggesting that immunocompromised patients with sepsis were more prone to anemia, coagulation disorders, and immunomodulatory therapy. Although there were no statistically significant differences between groups in case fatality rate, there were more patients in the control group with higher SOFA and APACHE II scores, were receiving vasoactive drugs and mechanical ventilation than in the immunocompromised group, implying that these patients were more prone to severe infection, mechanical ventilation, and shock.

**Table 1:** The basic clinical information of patients.

	Immunocompromised group (n = 92)	Control group (n = 216)	P-value
**Men (%)**	54 (58.69%)	141 (65.28%)	0.273
**Age (year)**	55 (48-66)	62.5 (49-71)	0.010
Clinical indicators			
SOFA score	5.00 (3.00-8.75)	6.00 (3.00-10.00)	0.226
APACHII score	14.00 (10.00-22.00)	16.00 (11.00-21.00)	0.497
Length of stay (day)	28.00 (19.00-39.00)	24.00 (14.00-37.00)	0.058
Admission to ICU (%)	35 (38.04%)	117 (54.17%)	0.010
Length of ICU stay (day)	13.00 (7.00-23.00)	17.00 (11.50-28.00)	0.083
Treatments			
Mechanical ventilation (%)	29 (31.52%)	105 (48.61%)	0.006
Duration of mechanical ventilation (day)	11 (4.5-20)	14 (8-22)	0.132
Renal replacement therapy (%)	15 (16.30%)	40 (18.52%)	0.642
Duration of renal replacement therapy (hour)	114.50 (39.50-262.75)	70.38 (41.25-167.75)	0.427
Vasoactive medications (%)	36 (39.13%)	108 (50.00%)	0.080
Glucocorticoids (%)	82 (89.13%)	127 (58.80%)	0.000
Blood products (%)	79 (85.87%)	155 (71.76%)	0.008
**Case fatality rate (%)**	28 (30.43%)	62 (28.70%)	0.760

### 3.2 Comparison of mNGS and culture’s diagnostic performance

**Figure 3:**
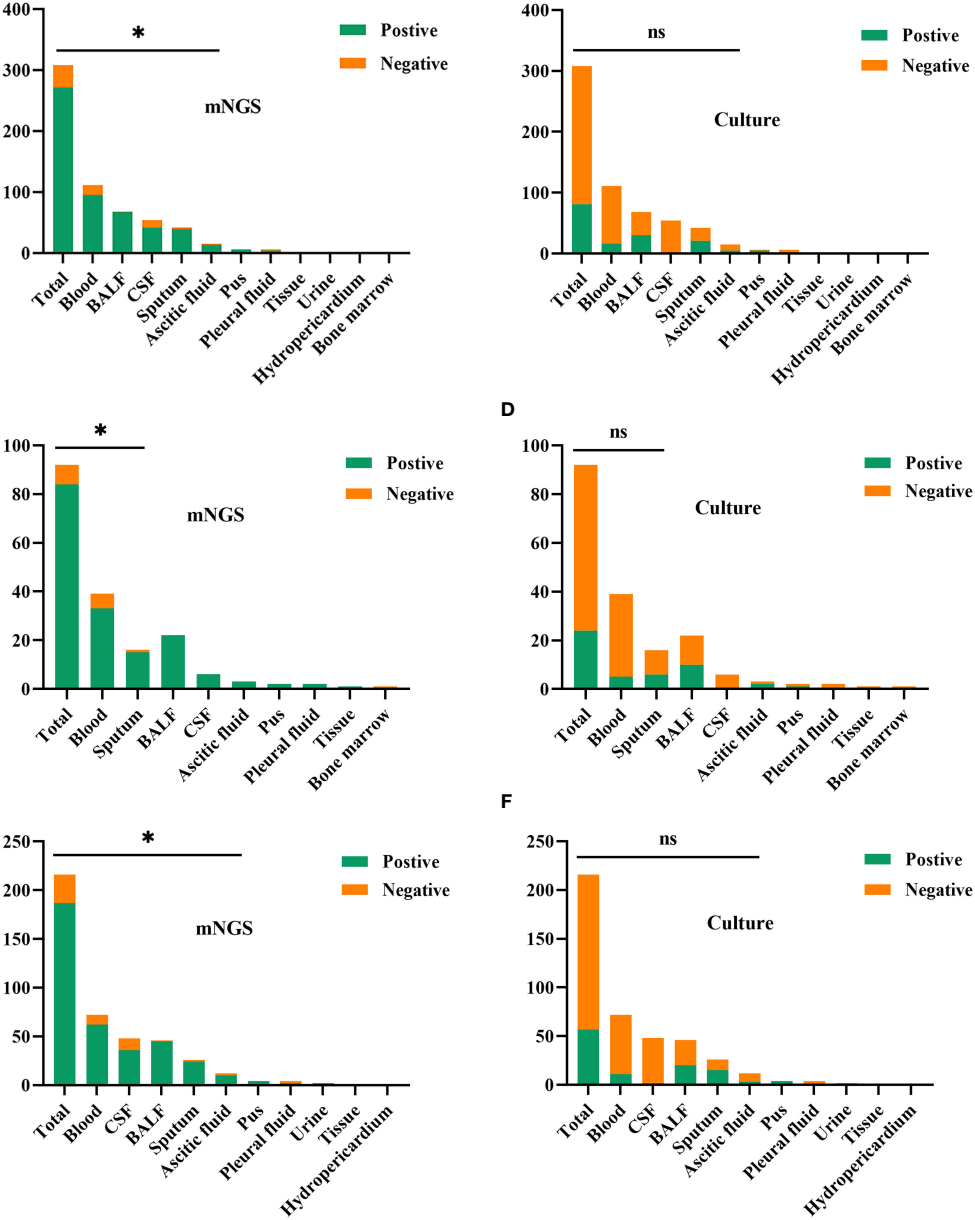
The comparison of positive rates between mNGS and culture method in sample types. **(A, B)** In all samples, there was considerably higher sensitivity in mNGS than those in the culture method in the types of blood (P < 0.001), BALF (P < 0.001), CSF (P < 0.001), sputum (P < 0.001) and ascitic fluid (P = 0.008). **(C, D)** In the immunocompromised group, mNGS results demonstrated more sensitivity than the culture method in the types of blood (P < 0.001), and sputum (P = 0.004). **(E, F)** In the control group, there was more sensitivity in mNGS than those in the culture method in the types of blood (P < 0.001), BALF (P < 0.001), CSF (P < 0.001), sputum (P = 0.004) and ascitic fluid (P = 0.016). mNGS, metagenomic next-generation sequencing; BALF, bronchoalveolar lavage fluid; CSF, cerebrospinal fluid. * p < 0.05; ns, no significant difference.

In this study, **Figure 3** shows the results of mNGS and culture methods. There were significant differences between the culture and mNGS method of all patients (P<0.001), of the immunocompromised group (P<0.001) and control group (P<0.001), in the chi-square test of positive rate. The results demonstrated that the sensitivity (positive number/number) was increased by roughly 62% in all samples (88.0% vs. 26.3%; P < 0.001), 65% in the immunocompromised group (91.3% vs. 26.1%; P < 0.001), and 60% in the control group (86.6% vs. 26.4%; P < 0.001) when mNGS was used in place of culture method. In all samples, there was considerably higher sensitivity in mNGS detection than those in the culture method in the types of blood (P < 0.001), BALF (P < 0.001), CSF (P < 0.001), sputum (P < 0.001) and ascitic fluid (P = 0.008). However, in the subtypes of pus, pleural fluid, tissue, urine, hydro pericardium, and bone marrow, there were no substantial differences in sensitivity between the two techniques owing to the limited sample size. The results of the control group were identical to those of the above (P < 0.001 in blood, P < 0.001 in BALF, P < 0.001 in CSF, P = 0.004 in sputum, P = 0.016 in ascitic fluid). Nevertheless, in the immunocompromised group, mNGS detection demonstrated considerably more sensitivity than the culture method in the types of blood (P < 0.001), and sputum (P =0.004). There was no apparent difference between the sensitivity of the two methods in the types of others due to the limited sample size.

**Figure 4:**
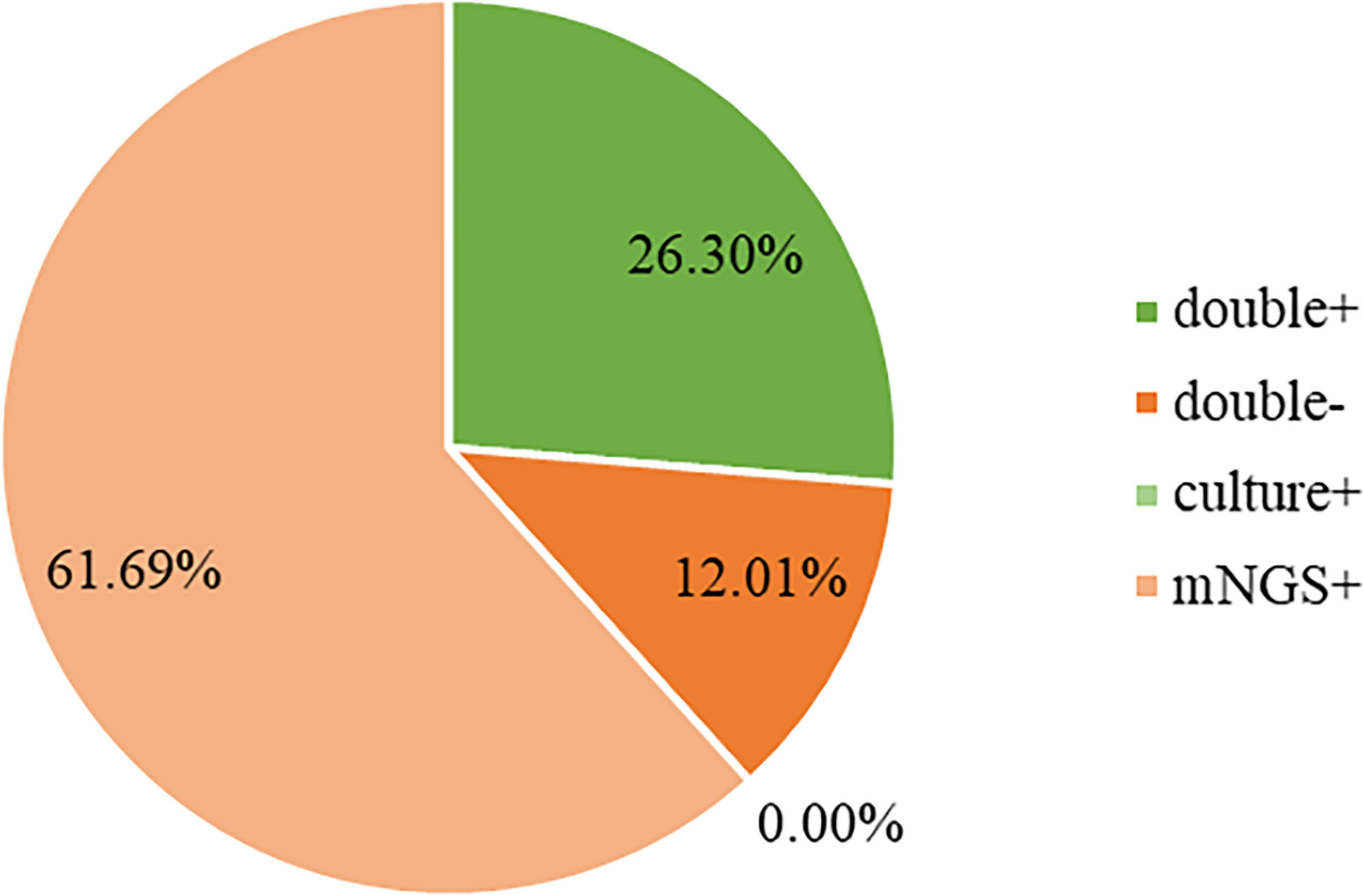
Concordance between mNGS and culture for pathogen detection. The pie chart demonstrated the positivity distribution of mNGS and culture for all samples. 61.69% were positive by mNGS, 0% by culture, 26.30% by both, and 12.01% were both negative. mNGS, metagenomic next-generation sequencing.

**Figure 5:**
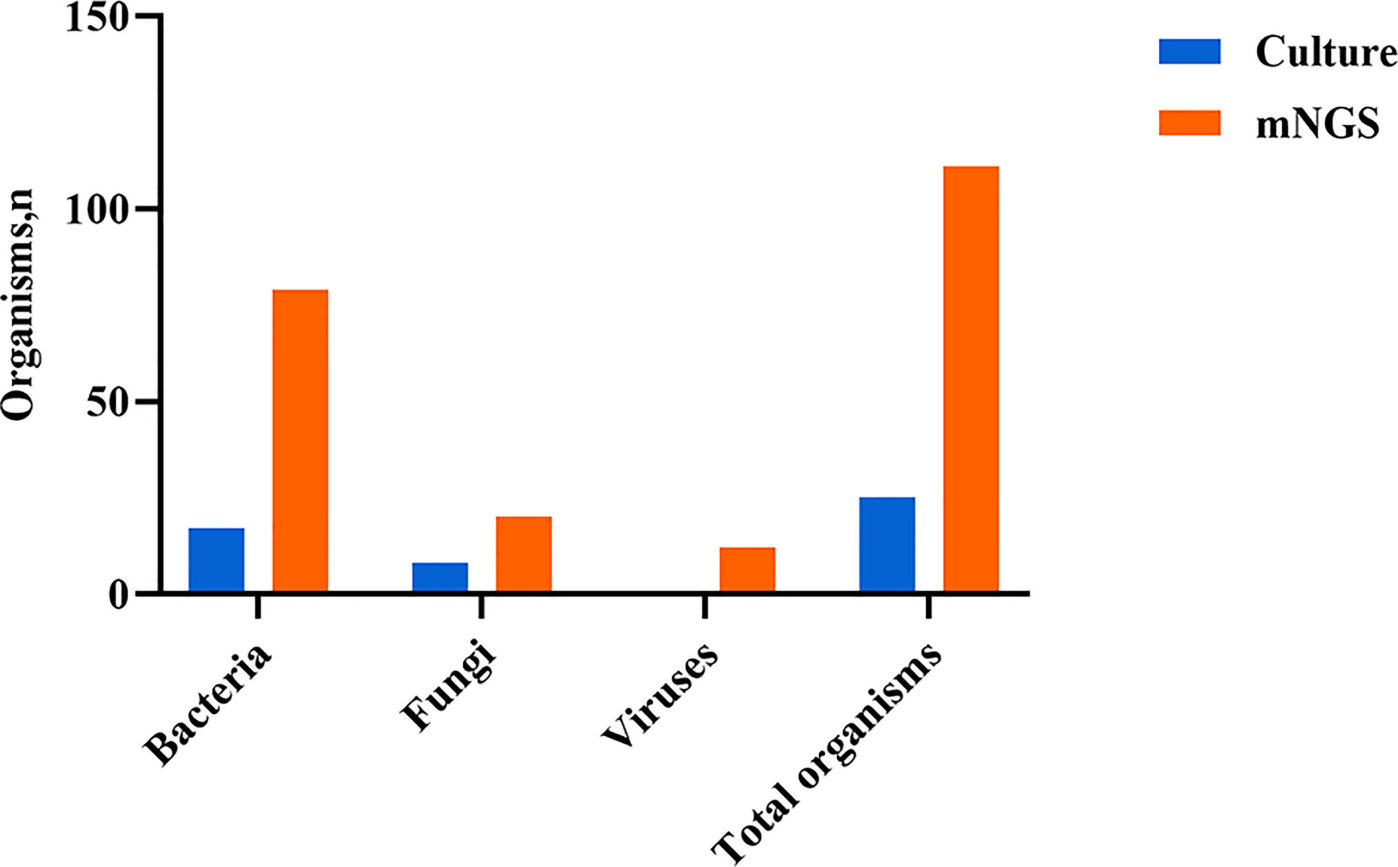
Type of organisms detected by mNGS compared with culture method. mNGS, metagenomic next-generation sequencing.

Both mNGS and culture method contribute to 81 of 308 (26.3%) cases of positive results and 37 of 308 (12.0%) cases of negative results in this study. Only mNGS detection was positive in 190 cases (61.7%), whereas only culture result was positive in 0 cases (0%). (**Figure 4**). The detection consequences were totally matched in 8 of 81 cases (overlap of all pathogens) and completely mismatched in 9 of 81 cases (overlap of no pathogen) in double-positive cases. The other 64 samples were characterized as “partly matched”, meaning that at least one but not all overlapped pathogens were founded in polymicrobial results. In 227 of 308 cases, mNGS detected one (n = 87), two (n = 57), or three or more (n = 46) organisms in each sample, while culture failed to identify any organism (**Table 2**). Conversely, in 37 cases where mNGS was negative, the culture method did not detect organisms. Consequently, compared with the culture method, mNGS detected more bacterial (79 vs 17), fungal (20 versus 8), and viral (12 versus 0) microorganisms (**Figure 5**).

**Table 2:** mNGS and culture results for each specimen: Comparison of microorganisms detected.

		Culture (n=25 microorganisms)			
		Negative	1	2	3+
mNGS (n=111 microorganisms)	Negative	37	0	0	0
	1	87	11	0	0
	2	57	14	0	0
	3+	46	49	7	0

**Figure 6:**
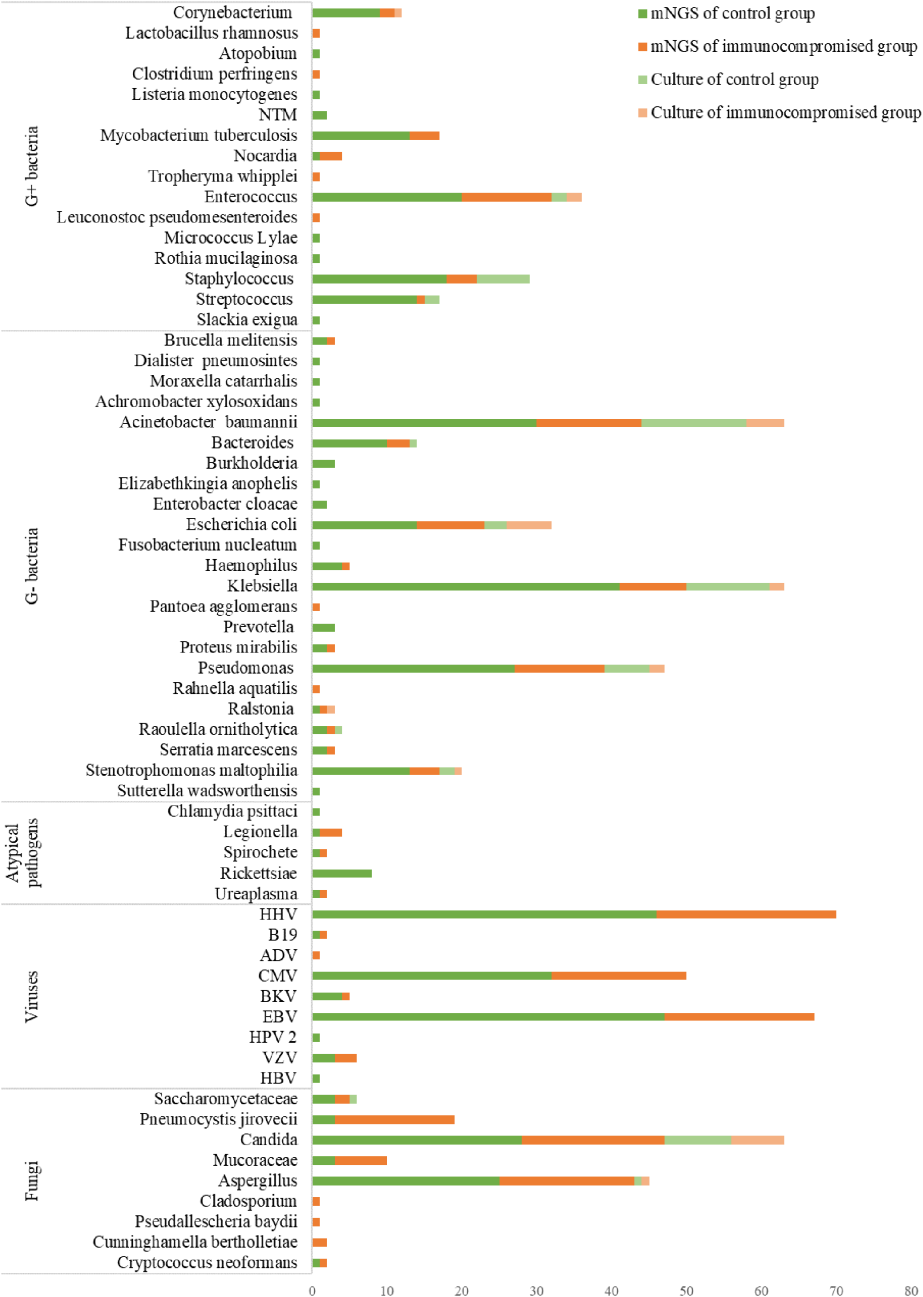
The distribution of detected pathogens of all patients by mNGS and culture method. The mNGS results were shown in dark green (control group) and orange (immunocompromised group), and the results of the culture method were shown in light green (control group) and orange (immunocompromised group). mNGS, metagenomic next-generation sequencing.

The pathogen identification outcomes of mNGS and culture are displayed in **Figure 6**. The most frequently identified bacteria were Klebsiella (n = 63) and Acinetobacter baumannii (n = 63) among the microbes identified using two methods, followed by Pseudomonas (n = 47), Enterococcus (n = 36), Escherichia coli (n = 32), Staphylococcus (n = 29), and Mycobacterium tuberculosis (n = 17). Additionally, there were Candida (n = 63) detected most frequently, followed by Aspergillus (n = 45) and Pneumocystis jirovecii (n = 19) in fungal organisms detected among the microbes.

There were 89 and 3 organisms detected respectively only by mNGS or culture. Microbes that were thought to induce sepsis and detected only by mNGS included Rickettsiae, Legionella, Nocardia, Mycobacterium tuberculosis, Mucoraceae, Pneumocystis jirovecii, and others. Furthermore, mNGS also detected viruses including HHV, EBV, and CMV, that were not identified by culture. There were only 3 organisms identified only by culture: Staphylococcus warneri, Candida lusitaniae, and Pichia ohmeri which were considered pathogens in this study. The followings are some plausible explanations for the missing species: either there was an extremely poor microbial loading of the specimen that was under the detection limit of mNGS or the microorganisms were excluded by the software as being part of the normal flora or environmental contaminants. There were substantial differences in the mNGS results between immunocompromised and control groups for Pneumocystis jirovecii(P < 0.001), Mucoraceae (P = 0.014), Klebsiella (P = 0.045).

### 3.3 Comparison of laboratory tests in immunocompromised and control group

In this study, we compared the laboratory results between the immunocompromised group and control group on the diagnosis day of sepsis using complete blood count, hepatorenal function, coagulation function, CRP, and PCT tests. There were statistically significant differences in hemocyte, hepatic and renal function, coagulation function, and procalcitonin between the immunocompromised group and control group according to the results (**Table 3**).

**Table 3:** The lab information of patients.

	Immunocompromised group (n = 92)	Control group (n = 216)	P-value
RBC (10^12^/L)	2.70 ± 0.87	3.54 ± 0.89	0.000
Hemoglobin (g/L)	82.99 ± 25.51	107.48 ± 26.56	0.000
WBC (10^9^/L)	5.90 (1.37-10.01)	9.46 (6.06-14.27)	0.000
Neutrophile (10^12^/L)	3.72 (0.54-7.94)	8.09 (4.61-12.49)	0.000
Lymphocyte (10^12^/L)	0.49 (0.28-1.05)	0.78 (0.45-1.21)	0.001
NLR	4.93 (0.67-15.17)	10.87 (5.36-19.64)	0.000
Eosinophil (10^9^/L)	0.00 (0.00-0.03)	0.01 (0.00-0.08)	0.003
PLT (10^9^/L)	74.00 (18.75-140.50)	143.50 (84.75-210.50)	0.000
ALT (U/L)	26.50 (17.00-74.00)	38.00 (21.25-90.50)	0.023
AST (U/L)	29.00 (19.25-53.50)	40.00 (24.25-85.00)	0.000
Tbil (umol/L)	12.95 (10.03-21.70)	15.60 (10.18-29.20)	0.208
Albumin (g/L)	31.85 (28.15-35.10)	32.70 (28.10-37.83)	0.213
BUN (mmol/L)	5.93 (4.15-12.53)	7.89 (4.96-15.88)	0.029
Creatinine (umol/L)	61.45 (43.78-91.70)	74.00 (53.20-146.58)	0.001
CRP (mg/L)	99.88 (45.44-156.49)	85.95 (32.08-158.45)	0.415
PCT (ug/L)	0.49 (0.12-2.00)	0.84 (0.21-5.18)	0.022
Fibrinogen (mg/dl)	3.81 (2.53-5.23)	4.31 (3.07-5.86)	0.035
PT (S)	14.20 (13.30-15.78)	14.90 (13.83-16.30)	0.010
APTT (S)	39.20 (33.35-45.85)	42.70 (36.60-48.00)	0.014
D- dimer (ug/L)	2.04 (0.96-5.11)	2.82 (1.41-6.59)	0.069
PTA (%)	82.10 (66.30-92.00)	77.50 (65.25-89.00)	0.137

### 3.4 Clinical effects of mNGS result on diagnosis and management

When mNGS results were examined for impacts on patient treatment, they were found to have a positive or no effect in 235 (76.3%) and 66 (21.4%) patients separately, while a negative effect was reported in 7 patients (2.3%). Positive mNGS results made for a definite diagnosis in the 235 positive effect samples. However, mNGS failed to identify any extra pathogens in 37 patients, and its consequences in 29 patients were considered contaminated or insubstantial, in the patients without effects (**Table 4**).

**Table 4:** Clinical effects of mNGS results on diagnosis and management.

Clinical effect	Role of mNGS result	Treatment changes owing to mNGS
Positive effect (n=235; 76.3%)	Contributed to definitive diagnosis (n=235; 76.3%)	Treatment adjusted without de-escalation (n =124; 40.3%)
		Antibiotic de-escalated (n = 61; 19.8%)
		Empirical treatment continued (n=50; 16.2%)
Negative effect (n =7; 2.3%)	False-positive result led to incorrect diagnosis (n=7; 2.3%)	Incorrect antibiotic treatment
No effect (n=66; 21.4%)	No additional pathogen detected (n=37; 12.0%)	No changes
	Results deemed false or insignificant (n=29; 9.4%)	

In the light of treatment, mNGS result caused a directly shift in management (185 of 235 positive effect patients) or a definite diagnosis that allowed the continued empirical treatment (50 of 235 positive effect patients). 61 of 185 patients, where the course of treatment was altered due to the mNGS results, issued in a de-escalation of antibiotics.

Notably, there were 7 cases of mNGS results with negative impact. In 5 of these cases, there were patients infected with RNA viruses (eg, SFTSV, Encephalitis B virus, hantaan virus), while mNGS only performed DNA detection without RNA detection in this study. The case detected Streptococcus suis covered by antibiotics before, which of the clinical impact was effectless but was improved after empirical antibiotic escalation. In addition, there was a case clinically diagnosed as tuberculous pleurisy, which reported Bacteroides, leading to not replacing with anti-tuberculosis drugs in time.

## 4 Discussion

Sepsis in immunocompromised patients has increased with the rise in the immunocompromised population, and the resultant septic shock and multiple organ dysfunction syndrome become the major reasons for mortality (Ramirez et al., 2020). Given the influence of immune function and underlying diseases, there are frequently conditional pathogens, uncommon pathogens, and mixed infections where these infections are characteristic of untypical clinical manifestations, rapid clinical progression, and severe death (Linden, 2009). Furthermore, the use of various anti-infective drugs results in a poor incidence of infection detection using conventional methods during the period of their diseases. Consequently, it is a clinical challenge to develop a timely and precise pathogen diagnosis. MNGS has been widely used to identify new pathogens and diagnose infections in humans since it analyzes the whole microbiome in patient samples (Lefterova et al., 2015). Thus, we investigated the utilization and differences between mNGS and the conventional culture method in adult sepsis, particularly in immunocompromised patients. In our study, samples from 308 patients with sepsis were collected and submitted to mNGS and conventional culture methods in a pairwise manner, where the samples included blood, BALF, CSF, sputum, pus, pleural fluid, tissue, urine, or bone marrow. Then, we thoroughly compared the clinical characteristics and consequences of the conventional culture method with mNGS, especially for immunocompromised patients.

The result showed that there were substantial differences in ages, receiving mechanical ventilation, glucocorticoids, and blood products, as well as admission to ICU, between the two groups. It was also suggested that the sensitivity of mNGS was greater than that of the conventional culture method. Additionally, a group of researchists observed that mNGS identified potential pathogens more quickly and sensitively than pathology and culture (Li et al., 2018). According to Miao’s study (Miao et al., 2018), it was observed that mNGS exhibited a higher sensitivity for the infectious disease diagnosis than the conventional culture method (50.7% vs. 35.2%) and that it had significant advantages for the identification of MTB, Nocardia, anaerobic bacteria, virus, and fungi in particular. The above findings were compatible with our study, which revealed that the sensitivity of mNGS was considerably greater than that of the culture method (88.0% vs 26.3%). The extreme sensitivity of mNGS may be attributed to a lengthy plasma survival time of pathogen DNA and the fact that antibiotic treatment has a minor impact on mNGS results, but a significant impact on conventional culture. In this research, the sensitivity of mNGS in samples of blood (P < 0.001), BALF (P < 0.001), CSF (P < 0.001), sputum (P < 0.001), and ascitic fluid (P = 0.008) was substantially higher than that of the culture method.

Our study emphasizes important situations in which mNGS enabled species-level pathogen identification, acting as the sole diagnostic tool or complementing standard results. One such field dealt with usual pathogens that cause infections but were not identified by culture method. This study also identified Nocardia species, Tropheryma whipplei, and anaerobic bacteria, which had poor yields by conventional culture but were detectable by mNGS.

The capability of mNGS to identify viruses that are currently not routinely examined in patients with sepsis in China is another advantage, where mNGS produced definitive diagnoses and improved patient cares in these cases. Most notably, the capacity to identify atypical bacteria, where microorganisms are undetectable in conventional culture methods and routine molecular testing is limited, is one of the critical obstacles that mNGS overcame for conventional culture methods. In this study, Rickettsiae, Legionella, and Chlamydia Psittaci were among the atypical bacteria detected through mNGS that were undetected through conventional culture methods. Legionella is a slow-growing bacterium that can cause extrapulmonary symptoms and severe community-acquired pneumonia, while also being useless to β-lactam antibiotic therapy, requiring a prompt diagnosis (Bradley and Bryan, 2019). Previously, the diagnosis has relied on a time-consuming culture necessitating a specialized medium and urine antigen testing that can barely detect a serogroup since molecular methods are neither generally accessible nor standardized (Bradley and Bryan, 2019). Currently, identification and molecular epidemiology investigations of Legionella have both shown promise when using direct sequencing from samples (Coscollá and González-Candelas, 2009). Psittacosis is brought on by the zoonotic pathogen Chlamydia psittaci, which is spread from birds to humans (Knittler et al., 2014). Although Chlamydia psittaci is a challenging organism to be detected, mNGS has been utilized to diagnose a case of sepsis and multi-organ failure caused by this organism previously (Zhang et al., 2020), emphasizing the importance of mNGS in this process. It is this fact that mNGS also identified Mycobacterium tuberculosis, which was undetectable by conventional culture methods in 17 cases, resulting in a clear diagnosis, further emphasizing the power of mNGS for species-level detection.

In addition, it is difficult that fungal infections to be identified by the conventional culture method. When compared with histology, mNGS has been reported to exhibit a higher specificity for detecting fungal pathogens in specimens and allows reliable species-level identification (Guarner and Brandt, 2011). It is sluggish and laborious to cultivate fungi, and a pure isolate with sporulation and distinguishing characteristics is required for their identification by macroscopic and microscopic morphology (Larkin et al., 2020). When the clinical microbiological method was negative, other research indicated the identification value of mNGS for Coccidioides and Aspergillus (Gu et al., 2021). In this research, mNGS (which identified Aspergillus in 36 patients) offered an excellent complement to culture (which identified Aspergillus in 2 patients), resulting in an efficient anti-fungal therapy. Except for molds with low recovery rates, other fungi, such as Pneumocystis Jirovecii and Mucoraceae, cannot be cultivated. Opportunistic pathogen Pneumocystis Jirovecii is a significant contributor to sepsis and mortality in immunocompromised people, which the main methods used for diagnosis are an insensitive fluorescent antibody test and, an unstandardized and widely unavailable Pneumocystis Jirovecii PCR (Sokulska et al., 2015). In our study, mNGS identified an extra 19 cases of Pneumocystis Jirovecii that conventional methods had missed (3 patients in the control group and 16 patients in the immunocompromised group). Pneumocystis jirovecii was also involved in 17 of the positive effect patients where mNGS detected multiple pathogens demanding different antibiotics, demonstrating the usefulness of mNGS in directing proper treatment coverage. Mucoraceae is also an opportunistic pathogen that disseminated infections caused by often involving the gastrointestinal tract, skin, lungs, orbit, paranasal sinuses, and central nervous system with lethality in immunocompromised patients. The diagnosis is based mainly on histopathology and a Mucoraceae PCR that has become less regularly used in clinical settings (Vallabhaneni and Mody, 2015), which can result in missed diagnoses. In our study, 10 patients of Mucoraceae (3 patients in the control group and 7 patients in the immunocompromised group) were detected through mNGS that were undetected through conventional culture methods, further emphasizing the role of mNGS in the diagnosis of this fungi.

Overall, in 235 (76.3%) patients, the mNGS results resulted in a positive effect, including the detection of more pathogens, a clear diagnosis, and assurance for antibiotic de-escalation therapy when a patient’s condition improves. There were only 7 cases of the negative effect, notably false-positive mNGS results that caused a misdiagnosis and inappropriate treatment. In those cases, there were five patients infected with RNA viruses which mNGS did not detect, suggesting that mNGS RNA testing should be considered if the patient was suspected of RNA virus infection. Noticeably, one case of Mycobacterium tuberculosis was missed by mNGS. Further investigation revealed that it was most likely that the mNGS DNA extraction procedure utilized in this study was insufficient since it did not include a committed step, which may have helped more thoroughly break down the mycobacteria’s cell wall. This serves as a reminder that regardless of downstream molecular methods used, specimen preprocessing optimization is crucial for organism recovery. Our research suggests the utilization of both conventional culture methods and mNGS to increase organism recovery and diagnostic precision. In one case, Streptococcus suis was identified through mNGS but the culture result came back negative. Antibiotics for Streptococcus suis were administered to the patient without success and eventually, the patient was improved after empirical antibiotic escalation. Furthermore, regardless of the rigorous algorithms incorporated into the bioinformatic software that ruled out potential contamination, mNGS consequences in 29 patients were considered as normal flora or contamination.

Some studies showed that it can be difficult to distinguish between colonization and human or environmental contamination because mNGS detects unbiased and broad-spectrum microbial DNA (de Goffau et al., 2018; Oechslin et al., 2018). Colonization and background contamination are specifically significant for samples with poorer pathogenic bacterial loads (Marsh et al., 2018). When employing the mNGS method solely, there is currently an unstandardized methodology for separating true etiological pathogens, colonization, and contamination. In order to avoid these problems, a rigorous bioinformatics threshold would be founded to rule out lab contaminants and shorten within-run spillover from highly positive specimens, as well as negative control may be incorporated at every stage of the specimen preparation and sequencing procedure (Salter et al., 2014). Additionally, clinical presentation and concurrent laboratory results including bacterial and fungal cultures should be taken into consideration when evaluating mNGS results. However, problems occur when mNGS results disagree with those of the gold-standard method. In this research, doctors utilized clinical judgments to determine whether to rely on the mNGS results, the conventional culture method results, or both. Even though unavoidable due to the continuously evolving techniques, the standardization of mNGS methods and analyses, and recognition of the main limits of mNGS, can start to address these difficulties. When numerous organisms are identified, transient microbial DNA, which may represent either dead or live microorganisms in the sample source area, could potentially complicate mNGS result and demand interpretations with great caution (Hogan et al., 2021). Even though some susceptibility information may be obtained by sequencing, the culture method is required for extensive susceptibility tests since pathogens exhibit varied resistance patterns that call for susceptibility testing (Metlay et al., 2019; Yang et al., 2019).

It is noteworthy that we discovered that mNGS had no impact on 66 (21.4%) patients, mostly because the majority of these patients had no additional pathogens detected by mNGS. It could partially account for the major drawback of this research, which was that mNGS only sequenced DNA, not RNA and that the samples were not entirely aligned with the infection sites. In this study, the second drawback was that the software utilized assumed that any microorganisms whose sequence reads fell below a predetermined threshold were contaminated and eliminated. It is the unsatisfied fact that real infections can develop owing to the complexity of infections and the uncertainty of function of normal flora species in some circumstances, even at low abundance (sequence reads). Therefore, the clinical situation must be taken into account while interpreting the results. It is crucial to note that a frequent issue with the mNGS method is background microbial contamination which might be filtered out partially through negative controls, so it calls for clinicians familiar with usual background microorganisms and better result explanations with clinical practices (Fan et al., 2018).

We systematically investigated sample type, sensitivity, and pathogen type between mNGS and conventional culture in this study. Based on this, we also analyzed the differences in sepsis between immunocompromised and control groups. The greatest deficiency of our study was its limited sample size, which led to a variety of results showing certain trends without attaining statistical significance regrettably. As a result, more patients need to be involved in future studies. The study had the additional drawback of not being randomized controlled. This study has certain drawbacks as a retrospective study, such as insufficient data and data accumulations beyond the researcher’s control. In addition, there are also numerous drawbacks in this study including limited generalizability resulting from a single-center study, absence of a gold-standard diagnostic comparator, and absence of detailed information on antibiotic usage.

## 5 Conclusion

In conclusion, mNGS showed better sensitivity than conventional culture, particularly in blood, BALF, CSF, and sputum samples, and there was a tendency for greater sensitivity in the detection of Pneumocystis jirovecii, Mucoraceae and Klebsiella in the immunocompromised group. In addition, mNGS offers a considerable impact in enhancing sepsis diagnosis and contributing to better patient treatment. As a consequence of our findings above, as well as additional advantages of mNGS such as rapid results, and less impact of antibiotic exposures, we believe that mNGS should be employed more frequently in the future for early pathogen diagnosis in sepsis patients, especially immunocompromised patients. Nonetheless, it will be challenging for clinicians to interpret mNGS results in guiding clinical therapy of infectious disorders.

## Data availability statement

The original contributions presented in the study are included in the article/supplementary material. Further inquiries can be directed to the corresponding author.

## Ethics statement

The studies involving human participants were reviewed and approved by The Medical Ethics Committee of the First Affiliated Hospital of Anhui Medical University. The patients/participants provided their written informed consent to participate in this study. Written informed consent was obtained from the individual(s) for the publication of any potentially identifiable images or data included in this article.

## Author contributions

HZ contributed to conceptualizing and designing this investigation. XL, SL, DZ, and MH collected and arranged the data. XL and HZ analyzed the data. XL wrote this article. All authors contributed to the article and approved the submitted version.
